# Research on Walking Gait Planning and Simulation of a Novel Hybrid Biped Robot

**DOI:** 10.3390/biomimetics8020258

**Published:** 2023-06-15

**Authors:** Peng Sun, Yunfei Gu, Haoyu Mao, Zhao Chen, Yanbiao Li

**Affiliations:** 1 College of Mechanical Engineering, Zhejiang University of Technology, Hangzhou 310023, China; sunpeng@zjut.edu.cn (P.S.);; 2 Key Laboratory of Special Purpose Equipment and Advanced Processing Technology, Ministry of Education and Zhejiang Province, Zhejiang University of Technology, Hangzhou 310023, China; 3 Huzhou Institute of Digital Economy and Technology, Zhejiang University of Technology, Huzhou 313000, China

**Keywords:** biped walking, gait planning, humanoid robotic legs, hybrid mechanism, linear inverted pendulum

## Abstract

A kinematics analysis of a new hybrid mechanical leg suitable for bipedal robots was carried out and the gait of the robot walking on flat ground was planned. Firstly, the kinematics of the hybrid mechanical leg were analyzed and the applicable relevant models were established. Secondly, based on the preliminary motion requirements, the inverted pendulum model was used to divide the robot walking into three stages for gait planning: mid-step, start and stop. In the three stages of robot walking, the forward and lateral robot centroid motion trajectories and the swinging leg joint trajectories were calculated. Finally, dynamic simulation software was used to simulate the virtual prototype of the robot, achieving its stable walking on flat ground in the virtual environment, and verifying the feasibility of the mechanism design and gait planning. This study provides a reference for the gait planning of hybrid mechanical legged bipedal robots and lays the foundation for further research on the robots involved in this thesis.

## 1. Introduction

The humanoid bipedal robot has the advantages of a small footprint and a large range of motion due to its discrete contact with the ground. In addition to ordinary planar operations, it can also adapt to complex and low regularity scenes. Since the birth of robotics research, it has been a hot topic in various fields of research. At present, most of the existing bipedal robots are series mechanisms, such as HRP-5P [1] from Japan’s National Institute of Advanced Industrial Science and Technology and HUBO [2] from Korea’s Institute of Advanced Science and Technology. This type of robot designed with series mechanical legs has a simple leg configuration, but it has shortcomings, such as a large inertia of mechanism motion and large accumulated errors between each motion pair. At present, a few hybrid design mechanical legs, such as WL-16 RIV [3] of Waseda University in Japan and LARM bot [4] of Cassino University in Italy, have problems; these include the large size of their mechanism, their complex structure, low simulation of motion performance and insufficient step length. Therefore, researchers began to study series parallel hybrid mechanisms to solve the problem of defects in a single mechanism, while combining the advantages of both to enable the feet to more realistically simulate the driving mode of various joint muscles in the human leg, thereby achieving the effect of steady walking. At present, the SAFFiR robot [5], THOR robot [6], and 7-degree of freedom hybrid mechanical legs have all simulated real walking situations well and have a high degree of bionics.

The biped robot is a strong coupling, nonlinear multi-degree of freedom complex dynamic system, usually using the D-H parameter method, screw theory and other methods for kinematics analysis [7]. The kinematics analysis of the hybrid mechanism is generally based on the Paden Kahan subproblem and screw theory. Different derivation methods present consistent results, but there are differences in the expression complexity, computational accuracy, and processing time. At present, the dynamic analysis of hybrid mechanisms requires selecting appropriate derivation methods based on the characteristics of different mechanisms.

There are generally three ways to plan the gait of a bipedal robot walking: offline planning, online planning, and online correction after offline planning [8,9,10]. Offline planning is based on the motion requirements of robots, which involves planning the motion trajectories of each joint in advance and is easy to implement. The online gait planning has strong flexibility and a very high level of environmental adaptation, but it requires the robot sensors and control systems to have an extremely high real-time accuracy, making it the most difficult to achieve [11]. Therefore, it is currently difficult to widely apply. In offline planning, there are, generally, the model method [12], the bionics method [13], intelligent algorithm generation [14], motion divergence component dynamics [15] and other planning methods. The model method is a commonly used method for the gait planning of bipedal robots, and constrains the corresponding motion conditions on a simplified model based on the characteristics of the robot and establishes gait planning based on motion requirements. It has strong universality and low computational difficulty [16]. However, due to the limited configuration design of hybrid mechanical legs, there are few reports on their gait planning research.

In this paper, the forward and inverse kinematics models of the new hybrid mechanical leg is established, based on screw theory and the POE formula. In the case of offline planning, the linear inverted pendulum model is used to plan the trajectories of different gait stages [17]. The calculation trajectory is derived using MATLAB software v.2023a, and the dynamics simulation is carried out using Adams software to verify the rationality of the mechanical leg configuration design, kinematics and dynamics models, and the robot gait planning, providing reference for the subsequent motor selection and optimization of the actual prototype.

## 2. Introduction of Biped Robot Model

**Figure 1:**
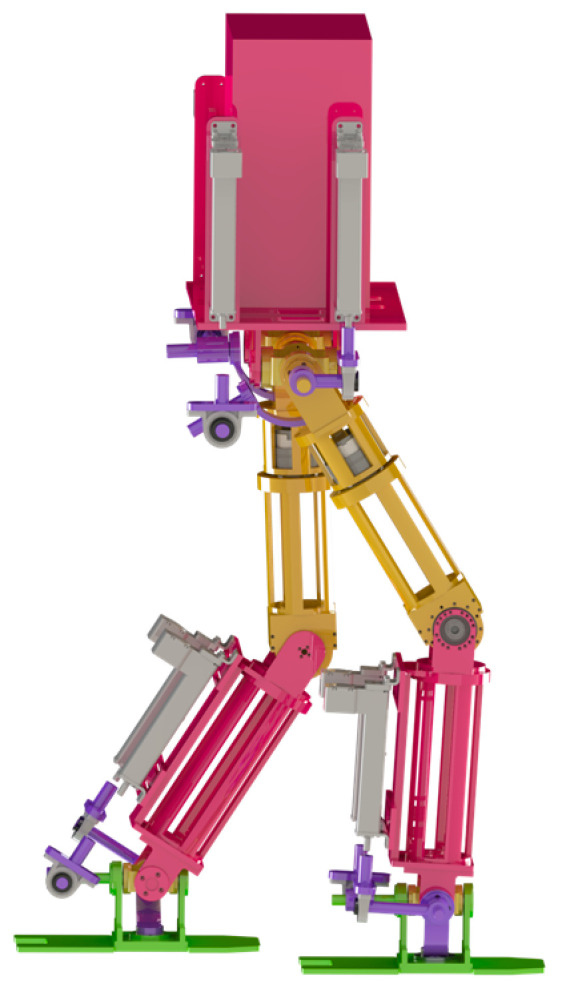
The diagram of the three-dimensional model of a bipedal robot.

The specific parameters of the bipedal robot are shown in Table 1 [18]. The overall structure of the bipedal robot involved in this article is shown in Figure 1, consisting of two parts: a lower limb platform composed of hybrid mechanical legs and a body platform. The hybrid mechanical leg is modeled after the real human leg joints, consisting of the hip joint, knee joint, and ankle joint. The hip joint parallel mechanism PRC–PRCR–RR mechanism comprises two spherical rotational degrees of freedom; the knee joint is composed of a rotating guide rod mechanism and an electric motor, with two degrees of freedom for rotation, and the ankle joint is isomorphic to the hip joint.

## 3. Kinematics Analysis

### 3.1. Establishment of Spatial Coordinate System

**Figure 2:**
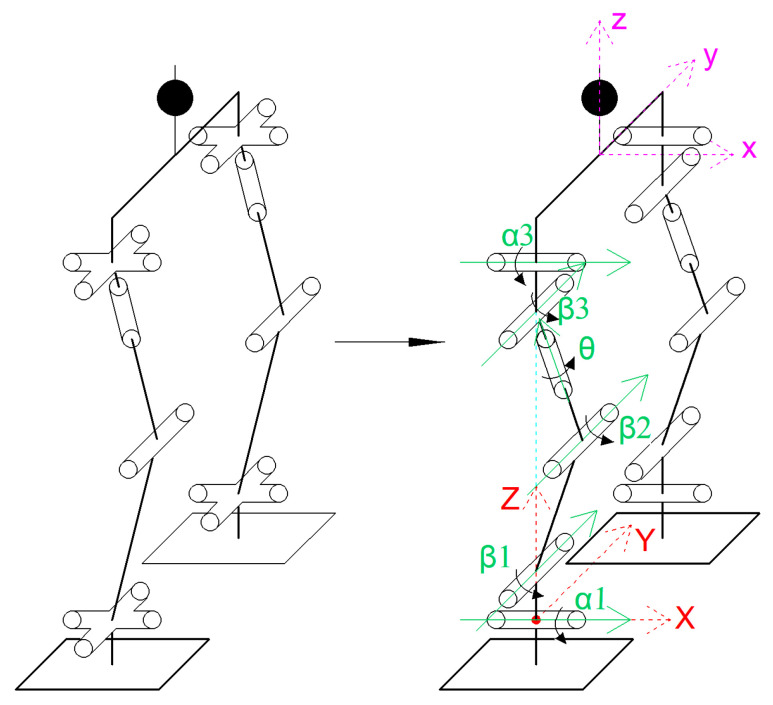
Equivalent series mechanical leg conversion diagram.

This article assumes that the right leg is the supporting leg in the initial state, and adopts a spatial coordinate system based on the supporting leg to establish a method. The number and properties of the degrees of freedom of each joint mechanism are maintained, and the converted equivalent series mechanical leg series mechanism is obtained, as shown in Figure 2. The hip joint mechanism and ankle joint are equivalent to 2-DOF series mechanisms, with the spatial coordinate system set at the center of the right-leg ankle joint and the auxiliary coordinate system set at the center of the two hip joints.

### 3.2. Forward Kinematics Solution of Hybrid Robot Leg

The forward kinematics model of the equivalent series mechanical leg is established using POE. Taking the support leg as an example, the forward kinematics model is established:

(1) $$\begin{array}{ll} g_{1} & =\exp\left(\overset{\wedge }{\xi_{X1}^{0}},\alpha_{1}\right)\cdot \exp\left(\overset{\wedge }{\xi_{Y1}^{0}},\beta_{1}\right)\cdot \exp\left(\overset{\wedge }{\xi_{Y2}^{0}},\beta_{2}\right)\cdot \exp\left(\overset{\wedge }{\xi_{Z3}^{0}},\theta \right)\cdot \\ & \exp\left(\overset{\wedge }{\xi_{Y3}^{0}},\beta_{3}\right)\cdot \exp\left(\overset{\wedge }{\xi_{X3}^{0}},\alpha_{3}\right)\cdot g_{0}\left(0\right) \end{array}$$

where $\xi_{X1}^{0}$, $\xi_{Y1}^{0}$, $\xi_{Y2}^{0}$, $\xi_{Z3}^{0}$, $\xi_{Y3}^{0}$, $\xi_{X3}^{0}$ is the initial rotational motion of each equivalent joint of the support leg.

Since there is redundancy in the parallel mechanism, *θ* should be specified as a constant value in order to obtain a unique solution for the input value of each joint. The value of *θ* is now set to 0, based on the requirement to move on level ground.

### 3.3. Inverse Kinematics Solution of Hybrid Robot Leg

By using the theoretical value of equivalent series joints as the output pose of the parallel mechanism platform, the inverse solution of the positions of each joint in the parallel mechanism can be obtained. Let the homogeneous coordinates of the hip joint rotation center ***o*** be ***p***, and the homogeneous coordinates of the ankle joint rotation center ***O*** be ***q***_1_. According to PK sub-problems 2 and 3, the forward kinematics model of the support leg can be deduced:

(2) $$\exp\left(\overset{\wedge }{\xi_{Y1}^{0}},\beta_{1}\right)\cdot \exp\left(\overset{\wedge }{\xi_{X1}^{0}},\alpha_{1}\right)\cdot \exp\left(\overset{\wedge }{\xi_{Z1}^{0}},\theta \right)\cdot \exp\left(\overset{\wedge }{\xi_{Y2}^{0}},\beta_{2}\right)\cdot p=g_{1}\cdot g_{0}{\left(0\right)}^{-1}\cdot p=g_{2}$$

Subtracting ***q***_1_ on both sides of the equation and taking the second-order norm on both sides, and then according to PK sub-problem 3, ***β***_1_, ***β***_2_, ***α***_1_ can be obtained:

(3) $$\left\{\begin{array}{l} \exp\left(\overset{\wedge }{\xi_{Y3}^{0}},\beta_{3}\right)\cdot \exp\left(\overset{\wedge }{\xi_{X3}^{0}},\alpha_{3}\right)=p_{2}\\ p_{2}=\exp\left(\overset{\wedge }{-\xi_{Y2}^{0}},\beta_{3}\right)\cdot \exp\left(\overset{\wedge }{-\xi_{Z1}^{0}},\theta \right)\cdot \exp\left(\overset{\wedge }{-\xi_{X1}^{0}},\alpha_{1}\right)\cdot \exp\left(\overset{\wedge }{\xi_{Y1}^{0}},\beta_{1}\right)\cdot g_{1}\cdot g_{0}{\left(0\right)}^{-1} \end{array}\right.$$

Let the homogeneous coordinates of a reference point be ***p***_3_. Multiply both sides of the equation by ***p***_3_, and then according to PK sub-problem 2, ***β***_3_ and ***α***_3_ can be solved.

At this point, the joint angle of the equivalent series mechanical leg can be solved, that is, the output angle of the hip joint motion platform, the output angle of the knee joint, and the output angle of the ankle joint motion platform can be obtained.

## 4. Planar Walking Gait Planning

Current gait planning methods are mainly applicable to mechanical legs comprising series or parallel mechanisms, and there is no extensive research on hybrid structures. Due to the concentration of the robot mass in the upper half, the leg mass can be ignored and considered as an inverted pendulum model centered on the ankle joint. By using an inverted pendulum model, inertia can be fully exploited, making the robot’s gait more natural and reasonable. At the same time, it can reduce the effect of the driving torque of the ankle joint on the walking stability of the robot [19,20]. The smaller the torque of the ankle joint, the larger the stability margin of the robot’s gait and the more stable the gait. The ankle joint has two degrees of freedom in motion, so planning around it requires finding the trajectories of the forward and lateral gait separately. The forward gait includes the trajectories of three mechanisms, hip, knee and ankle, while the lateral gait includes the trajectories of two mechanisms, hip and ankle.

A complete gait pattern comprises an initial phase, an intermediate phase and a stopping phase. The mid-stage is periodic, while the start and stop stages require the speed of the swinging leg during lifting and falling to not be too high, in order to avoid excessive impact force on contact with the ground, causing jerking and affecting stability. Therefore, the gait is planned in three phases.

### 4.1. Mid-Step Gait Planning Based on Linear Inverted Pendulum

During robot movement, it can be simplified as a pendulum rod centered around the ankle joint of the supporting leg, ignoring the mass of the swinging leg. In a linear inverted pendulum, the center of mass does not move in the *Z* direction and always moves parallel to the ground. Therefore, only the coordinate changes in the center of mass in the *X* and *Y* directions can be solved.

#### 4.1.1. Forward Gait Planning of The Centroid

**Figure 3:**
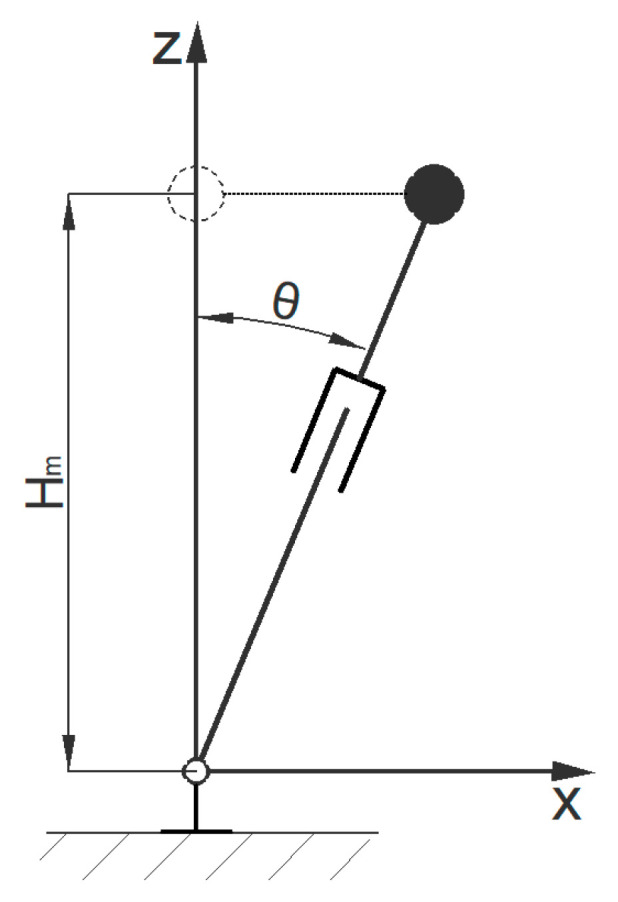
Simplified inverted pendulum models for the forward gaits.

The forward gait model is shown in Figure 3.

The equation of motion for an inverted pendulum is as follows:

(4) $$\ddot{\theta }-\frac{g}{l}\sin(\theta )=0$$

The general solution of this equation is as follows:

(5) $$\theta \left(t\right)=c_{1}e^{-kt}+c_{2}e^{kt}$$

According to the model, the relationship equation between the center of gravity coordinate and the length of the inverted pendulum can be obtained:

(6) x=lsinθ

The single-step length in the periodic gait is set to ***s***, the time required for a single step is set to ***T_s_***, and the bipedal support time is set to ***T_d_*** = ***T_s_***/4. The height of the overall center of mass relative to the ankle joint is ***h***. During the walking process of a bipedal robot, the motion of the legs is periodic and symmetrical; therefore, there are motion constraints:

(7) $$\left\{\begin{array}{l} \dot{x}(t)>0,\;\dot{\theta }(t)>0\\ x\left(T_{s}\right)-x(0)=s \end{array}\right.$$

Assuming that the moment when the centroid passes through the vertical axis is ***T_m_***, the following can be obtained:

(8) $$\theta \left(t_{m}\right)=\theta_{m}=0$$

Based on these equations, the equations for the trajectory of the center of mass in the *X* direction are solved.

#### 4.1.2. Lateral Gait Planning of The Centroid

**Figure 4:**
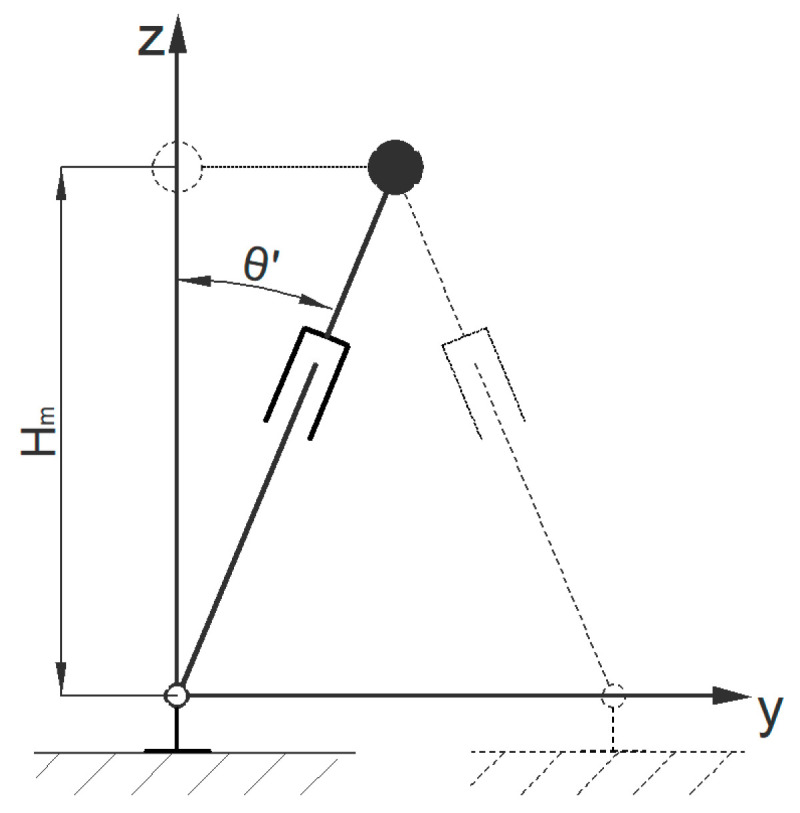
Simplified inverted pendulum models for the lateral gaits.

In a lateral gait, there are alternating transformations in the inverted pendulum model. When the swinging leg and the supporting leg are in the same plane, the center of mass passes directly above the supporting leg, and it is specified that the lateral motion system time *t* = 0 (when the lateral model system time is 0, there is a difference of one *T_s_*/2 from the forward model system time of zero). In lateral motion, the velocity of the center of mass is 0. When the system time *t* = *T_s_*/2, the inverted pendulum model is in the state shown in Figure 4.

According to the geometric relationship of the model, the motion constraints are set as follows:

(9) $$\left\{\begin{array}{l} {\dot{\theta }}_{m}^{\prime }(0)=0\\ \theta_{m}^{\prime }(T_{s}/2)=\arcsin\left(\frac{W_{d}/2}{H_{m}}\right) \end{array}\right.$$

When the system time is [*T_s_*/2, *T*], the inverted pendulum model changes from the position shown in Figure 4, and the supporting leg and swinging leg alternate. The displacement change in the center of mass is symmetrical with the motion state within [0, *T_s_*/2].

The equations of motion for the lateral model can be obtained from Formula (4) in the same way. Based on the equations of motion with the above constraints, the equation of the trajectory of the center of mass in the *Y* direction can be solved.

#### 4.1.3. Forward Gait Planning of The Swinging Leg

The trajectory planning of the swinging legs is usually represented by the trajectory of the ankle position. Landing on the foot will cause some degree of impact and affect stability. Polynomial interpolation is used in the calculation to avoid the risk of impact and to ensure a smooth velocity change curve.

In the mid-stride phase, there is an alternation between the single and bipedal support. In general, the swinging leg lands and the start of the mid-stride cycle is when it enters bipedal support. At this point, the forward gait system time *t* = 0 is taken as the bipedal support phase time Td of the single-step cycle. Due to the symmetry of the leg movements, it is only necessary to plan a single-step gait to obtain a full-cycle gait. An *XYZ* coordinate system is set up at the projection point of the ankle joint of the support leg, with the *X* axis as the forward direction and the *Z* axis vertically upwards.

During the bipedal support period, if the position of the swinging leg remains unchanged in the *X* direction, the motion constraints within the system time [0, *T_d_*] can be obtained as follows:

(10) $$x_{a}(t)=-s,t\in \left[0,T_{d}\right]$$

During the single-leg support period, i.e., within the system time [*T_d_*, *T_s_*], according to the motion conditions and positional requirements of the swinging leg, its motion constraints can be expressed as follows:

(11) $$\left\{\begin{array}{l} x_{a}\left(T_{d}\right)=-s\\ x_{a}(T)=s\\ {\dot{x}}_{a}\left(T_{d}\right)=0\\ {\dot{x}}_{a}(T)=0\\ {\ddot{x}}_{a}\left(T_{d}\right)=0\\ {\ddot{x}}_{a}(T)=0 \end{array}\right.,t\in \left[T_{d},T_{s}\right]$$

Based on the above equation, the equation for the trajectory of *x* can be solved as follows:

(12) $$\left\{\begin{array}{r} x_{a}(t)=-s,t\in \left[0,T_{d}\right]\\ x_{a}(t)=k_{0}+k_{1}t+k_{2}t^{2}+k_{3}t^{3}+k_{4}t^{4}+k_{5}t^{5},t\in \left[T_{d},T_{s}\right] \end{array}\right.$$

In the *Z* direction, the sole must reach its highest point in the middle of a single step. Due to the symmetry of the single-step motion trajectory in the *Z* direction, the trajectory during the single-step support period is divided into two stages for planning, namely lifting and falling, from the moment at which the swinging leg passes the highest point.

During the bipedal support period, the swinging leg does not change in the *Z* direction, i.e., within the system time [0, *T_d_*], the following relationship can be obtained:

(13) $$z=h_{foot},\;t\in \left[0,T_{d}\right]$$

When the swinging leg is in the lifting process, i.e., [*T_d_*, *T_m_*], the motion constraints can be obtained as follows:

(14) $$\left\{\begin{array}{l} z_{a}\left(T_{d}\right)=h_{foot}\\ z_{a}\left(T_{m}\right)=h_{foot}+H\\ {\dot{z}}_{a}\left(T_{d}\right)=0\\ {\dot{z}}_{a}\left(T_{m}\right)=0\\ {\ddot{z}}_{a}\left(T_{d}\right)=0\\ {\ddot{z}}_{a}\left(T_{m}\right)=0 \end{array}\right.,t\in \left[T_{d},T_{m}\right]$$

When the motion constraint relationship of the swinging leg is opposite during the falling process, the trajectory of the change in the Z-coordinate of the swinging leg can be obtained:

(15) $$\left\{\begin{array}{r} z_{a}(t)=h_{foot},\;t\in \left[0,T_{d}\right]\\ z_{a}(t)=k_{0}+k_{1}t+k_{2}t^{2}+k_{3}t^{3}+k_{4}t^{4}+k_{5}t^{5},t\in \left[T_{d},T_{m}\right]\\ z_{a}(t)=k_{0}^{\prime }+k_{1}^{\prime }t+k_{2}^{\prime }t^{2}+k_{3}^{\prime }t^{3}+k_{4}^{\prime }t^{4}+k_{5}^{\prime }t^{5},t\in \left[T_{m},T_{s}\right] \end{array}\right.$$

#### 4.1.4. Calculation of Joint Angles Based on a Generalized Coordinate System

**Figure 5:**
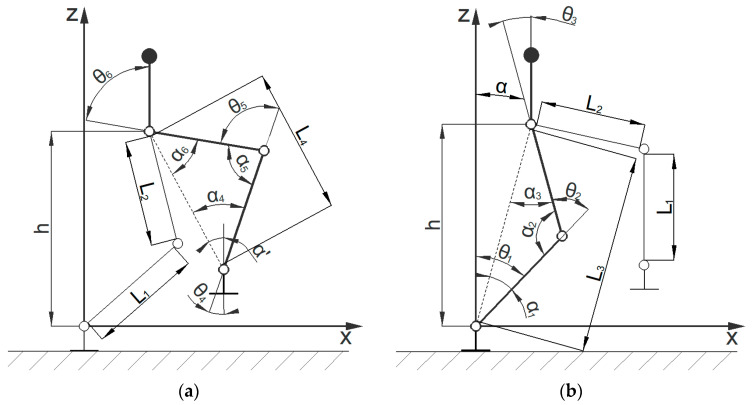
Simplified joint model of the robot during the mid-step gait. (**a**) Simplified joint model of the swinging leg and indication of each joint angle; (**b**) simplified joint model of the support leg and indication of each joint angle.

After obtaining the centroid motion trajectory and swinging leg motion trajectory, the angle changes in each joint can be obtained based on generalized coordinates. Based on the structural characteristics of the bipedal robot, the state of the legs at a certain moment in the mid-step gait is simplified to obtain the angle states of each joint between the swinging leg and the supporting leg, as shown in Figure 5.

Given the centroid trajectory (*x_m_*, *z_m_*), let the distance from the centroid of the bipedal robot to the hip joint be *l_m_*, with a thigh length of ***l***_2_, and a calf length of ***l***_1_. According to the geometric relationship in the figure above, the following equation can be obtained:

(16) $$\left\{\begin{array}{l} h=z_{m}-l_{m}\\ L_{3}=\sqrt{x_{m}^{2}+h^{2}}\\ \alpha =\arctan\left(\frac{x_{m}}{h}\right)\\ \alpha_{1}=\arccos\left(\frac{L_{1}^{2}+L_{3}^{2}-L_{2}^{2}}{2L_{1}L_{3}}\right)\\ \alpha_{2}=\arccos\left(\frac{L_{1}^{2}+L_{2}^{2}-L_{3}^{2}}{2L_{1}L_{2}}\right)\\ \alpha_{3}=\pi -\left(\alpha_{2}+\alpha_{1}\right) \end{array}\right.$$

The angle relationship of each joint can be obtained via the generalized coordinate system, as follows:

(17) $$\left\{\begin{array}{l} \theta_{1}=\alpha_{1}+\alpha \\ \theta_{2}=\pi -\alpha_{2}\\ \theta_{3}=\alpha_{3}-\alpha \end{array}\right.$$

Similarly, based on the relationship between the ankle joint motion trajectory (*x*, *z*) and the centroid motion trajectory (*x_m_*, *z_m_*), the following equation can be derived:

(18) $$\left\{\begin{array}{l} \alpha^{\prime }=\arctan\left(\frac{x-x_{m}}{z_{m}-l_{m}-z-h_{foot}}\right)\\ L_{4}=\sqrt{{\left(x-x_{m}\right)}^{2}+{\left(z_{m}-l_{m}-z-h_{foot}\right)}^{2}} \end{array}\right.$$

The trajectory of each joint of the swinging leg can be derived as follows:

(19) $$\left\{\begin{array}{l} \theta_{3}=\alpha_{3}-\alpha^{\prime }\\ \theta_{4}=\pi -\alpha_{3}\\ \theta_{6}=\alpha +\alpha^{\prime } \end{array}\right.$$

**Figure 6:**
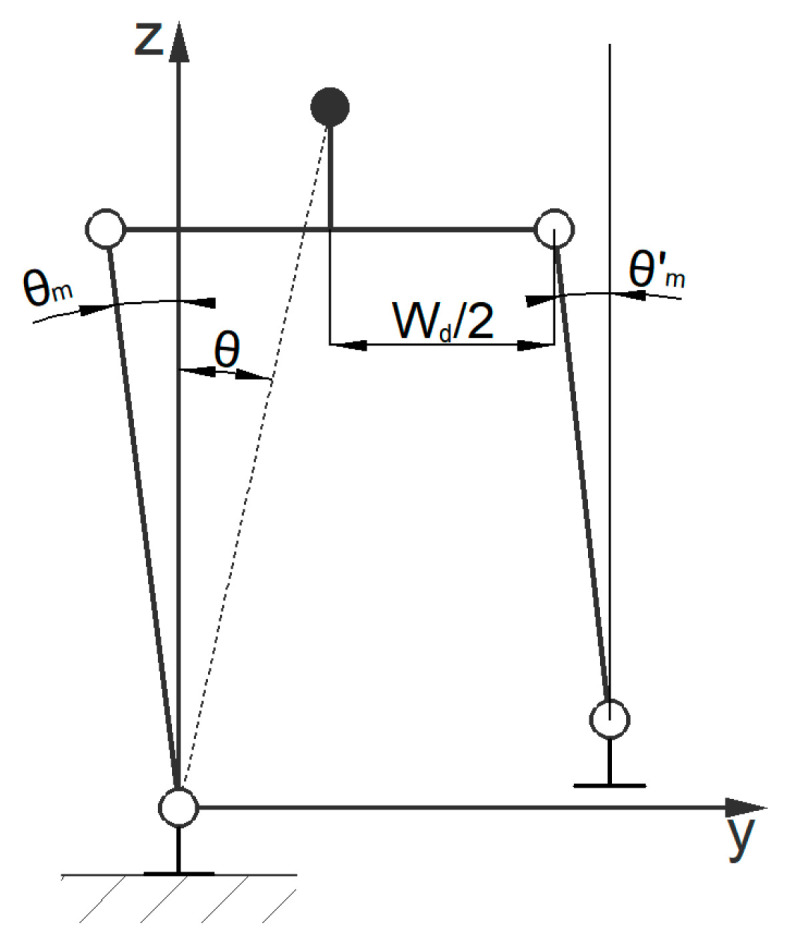
Simplified joint model for the lateral gait indication of each joint angle.

In a lateral gait, the robot’s upper body and foot soles are always parallel to the ground, and the knee joints have no lateral motion degrees of freedom, which can simplify the robot model, as shown in Figure 6.

The generalized coordinate trajectory of the center of mass is (*y_m_*, *z_m_*), the vertical distance between the center of mass and the hip joint is *l_m_*, and the distance between the centers of the two soles of the bipedal robot is *W_d_*. Based on the geometric relationship, the following equation can be obtained:

(20) $$\theta_{m}(t)=\arcsin\left(\frac{W_{d}/2-y_{m}}{z_{m}-l_{m}}\right)$$

#### 4.1.5. Mid-Step Gait Planning Results

**Figure 7:**
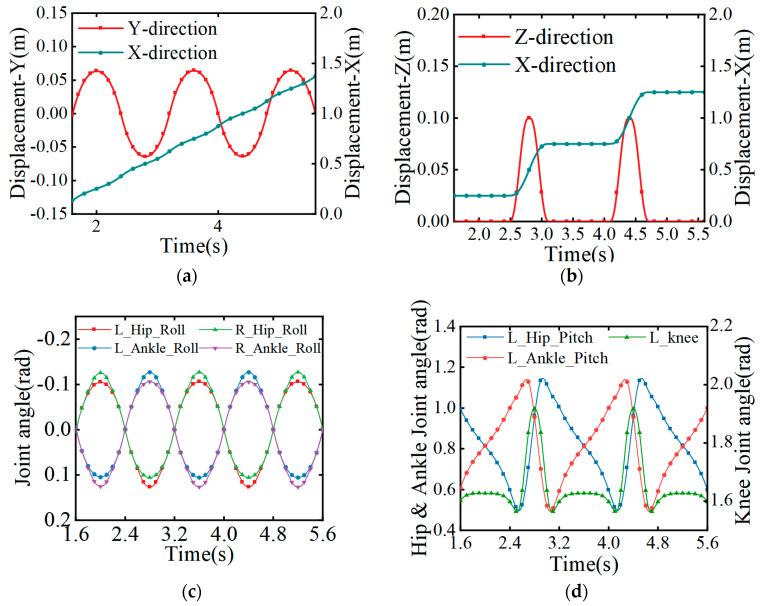
Movement trajectory and angular changes in the parts during the mid-step gait. (**a**) The motion trajectory of the center of mass; (**b**) the motion trajectory of the swinging leg; (**c**) the change in the angle of each joint of the swinging leg; (**d**) the change in the angle of each joint of the support leg.

In summary, the gait of a bipedal robot in a periodic mid-step gait can be obtained as, shown in Figure 7.

### 4.2. Starting Gait Planning

#### 4.2.1. Trajectory Planning of The Centroid

**Figure 8:**
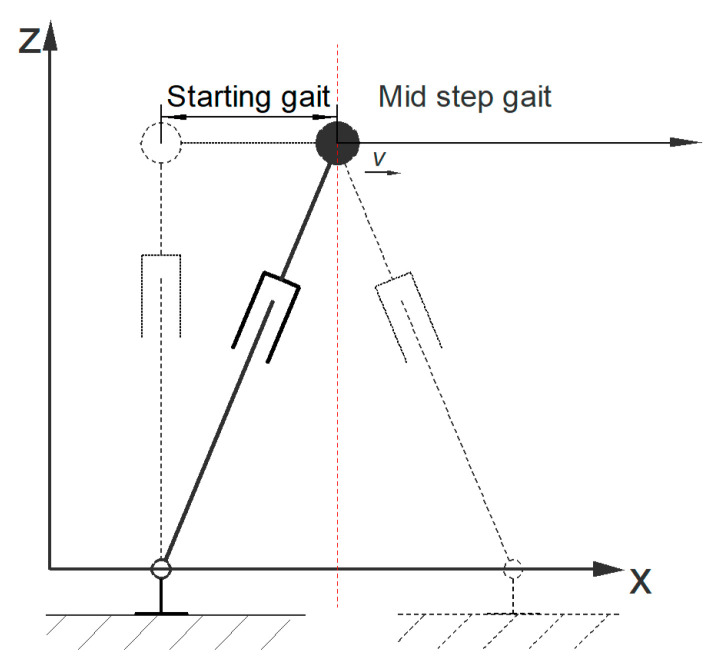
The schematic diagram shows the posture of the inverted pendulum model during the transition from the starting stage to the mid-step stage.

The starting stage is a transition state from the static to the intermediate stage, from static balance with zero displacement and zero speed to dynamic balance. The attitude during the transition is shown in Figure 8. According to the travel requirements of a linear inverted pendulum, and considering the energy loss caused by joint gaps, accuracy, and other factors, it is necessary to reduce the center of gravity to allow the robot enough space to adjust its joint angles during travel. Due to the differences between the robot’s motion model and the inverted pendulum model during this process, dynamic relationships are used to plan the starting stage.

In the *X* direction, the motion of the center of mass deviates significantly and there is a possibility of instability. Therefore, in order to ensure a smooth connection between position and speed, the following motion constraints exist for the *X* direction:

(21) $$\left\{\begin{array}{l} x(0)=0\\ \dot{x}(0)=0\\ x\left(T_{s}\right)=s/2\\ \dot{x}\left(T_{s}\right)=v_{x} \end{array}\right.$$

In order to maintain the balance of the motion process, when the deviation in the projection point of the center of gravity increases, the acceleration should also increase. Therefore, the acceleration in the *X* direction is planned by using a quadratic function. When *t* = *T_s_*, the center of gravity deviates the most severely, thus assuming the following:

(22) $$x_{zmp}=0,\;t=T_{s}$$

According to the ZMP calculation formula, the following equation can be obtained:

(23) $$\frac{mgx-m\ddot{x}z}{mg}=x\left(T_{s}\right)-\frac{\ddot{x}\left(T_{s}\right)z}{g}=0$$

Based on the above equation, the equation for the trajectory of *X* can be solved as follows:

(24) $$x(t)=k_{0}t^{2}+k_{1}t^{3}+k_{2}t^{4}$$

The lateral model motion state in the start phase also has similarities to the mid-step phase, so it is only necessary to use a correction function for the lateral joint angle change in the mid-step phase.

#### 4.2.2. Trajectory Planning of Swinging Leg

The motion posture of the swinging leg during the starting stage is similar to that of the mid-step stage, with an initial bipedal support period. The difference is that the initial position of the swinging leg is different, and the starting point of the system time is different. Therefore, within the system time [0, *T_d_*], there are the following motion constraints:

(25) $$x_{a}(t)=0,\;t\in \left[0,T_{d}\right]$$

Within the system time [*T_d_*, *T_s_*], there are the following motion constraints:

(26) $$\left\{\begin{array}{l} x_{a}\left(T_{d}\right)=0\\ x_{a}(T_{s})=s\\ {\dot{x}}_{a}\left(T_{d}\right)=0\\ {\dot{x}}_{a}(T_{s})=0\\ {\ddot{x}}_{a}\left(T_{d}\right)=0\\ {\ddot{x}}_{a}(T_{s})=0 \end{array}\;,\left[T_{d},T_{s}\right]\right.$$

Based on the above equation, the equation for the trajectory of *X* can be solved as follows:

(27) $$\left\{\begin{array}{cc} x_{a}(t)=-s & t\in \left[0,T_{d}\right]\\ x_{a}(t)=k_{0}+k_{1}t+k_{2}t^{2}+k_{3}t^{3}+k_{4}t^{4}+k_{5}t^{5} & t\in \left[T_{d},T\right] \end{array}\right.$$

**Figure 9:**
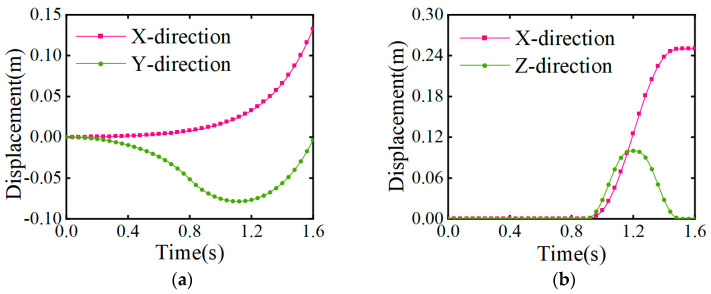
Change in trajectory during the starting phase. (**a**) The motion trajectory of the center of mass; (**b**) the motion trajectory of the swinging leg.

The change in the trajectory of the swinging leg in the *Z* direction during the starting phase can be obtained via polynomial interpolation (see Section 4.1.3). In summary, the center of mass and the swinging leg motion trajectory during the starting stage can be obtained, as shown in Figure 9.

**Figure 10:**
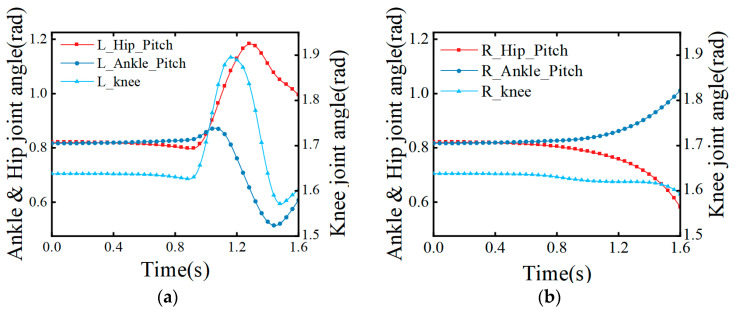
Movement trajectory and angular changes of the parts in the mid-step gait. (**a**) Change in the angle of each joint of the swinging leg; (**b**) change in the angle of each joint of the support leg.

According to the generalized coordinate trajectory calculation of the joints of the support leg and swinging leg in the forward gait mentioned earlier, the motion trajectory of the joint angles of the support leg and swinging leg in the starting stage can be obtained, as shown in Figure 10.

### 4.3. Stopping Gait Planning

**Figure 11:**
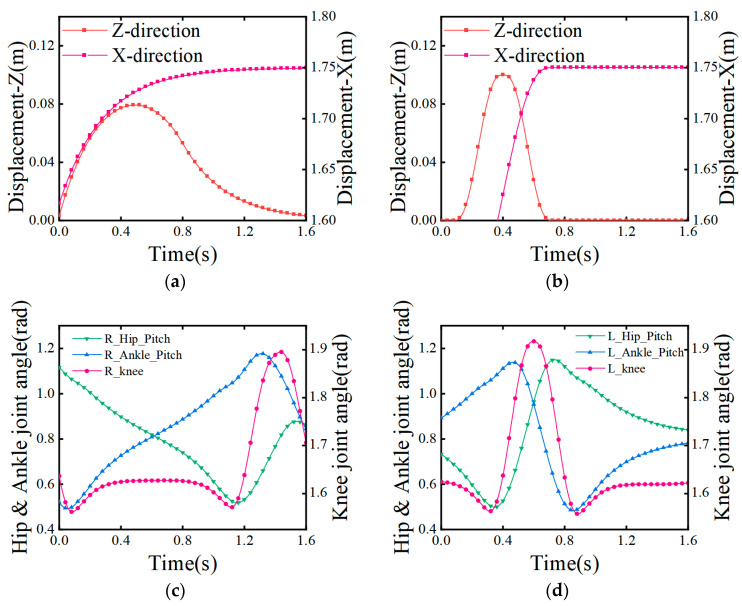
Movement trajectory and angular changes in the parts during the stopping gait. (**a**) The motion trajectory of the center of mass; (**b**) the motion trajectory of the swinging leg; (**c**) change in the angle of each joint of the swinging leg; (**d**) change in the angle of each joint of the support leg.

The motion characteristics of the stopping stage are similar to those of the starting stage, with the difference being that their initial and ending states alternate with each other. Therefore, based on the symmetry of bipedal walking, the motion constraints of the stopping stage can be inferred from the gait of the starting stage. From this, the trajectory of the center of mass during the stopping stage can be obtained, as shown in Figure 11.

## 5. Results and Discussion

**Figure 12:**
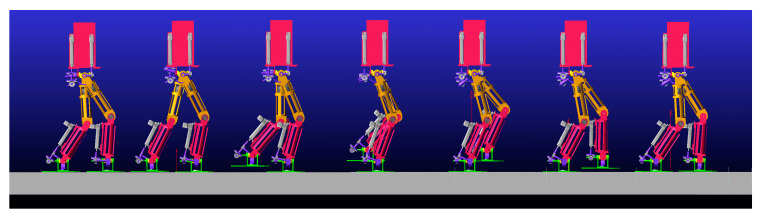
Sequence diagram of robot’s walking posture on flat ground.

As shown in Figure 10, simulation experiments were carried out in Adams software for a bipedal robot walking on flat ground. According to the design requirements, the overall mass of the robot was 100 kg. The two simulation experiments were conducted by importing the discrete points of the robot joint angle trajectory curve via simulation software. The first simulation had a single-step length of *s* = 0.25 m, a single-step period of *t* = 0.8 s, and a simulation end time of 8 s. The second simulation had a single-step length of 0.5 m, while the rest remained unchanged. The robot walking sequence is shown in Figure 12.

**Figure 13:**
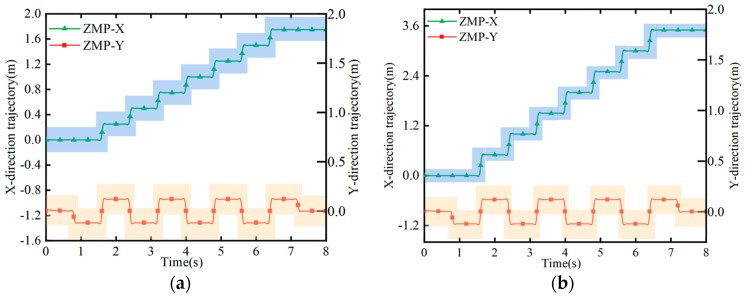
ZMP trajectory tracking. The blue and yellow areas represent the allowable range of the ZMP coordinate values within the support domain. (**a**) Trajectory at 0.25 m step distance; (**b**) trajectory at 0.5 m step distance.

According to the simulation, the actual ZMP trajectory was obtained, as shown in Figure 13. ZMP is a commonly used method for determining the stable state of the walking motion of bipedal robots. The definition of ZMP can be obtained from the literature [21,22]. Usually, when the ZMP is in the sole support region of a bipedal robot, the motion state of the robot can be considered stable. In theory, the trajectory of the ZMP is stepwise. However, according to the observation in the figure, the actual ZMP curve formed a smooth curve. In actual walking, if the ZMP is always in the position of the bipedal support domain, the robot’s walking is stable and will not overturn [23].

**Figure 14:**
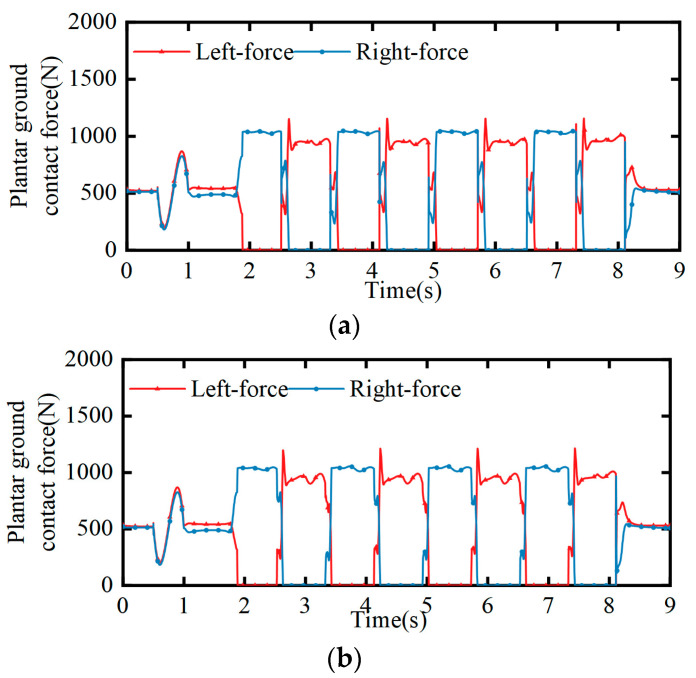
Changes in the contact force between the soles of both feet. (**a**) Contact force at 0.25 m step distance; (**b**) contact force at 0.5 m step distance.

At the same time, the change in the contact force between the soles of the bipedal robot and the ground during the simulation process was obtained, as shown in Figure 14. The change in the contact force was measured before the bipedal robot started squatting, and a slow squatting motion was completed within 0–1 s in order to achieve the required position of the center of mass. The contact force changed due to the acceleration and deceleration process of the center of mass. Within 1–2.6 s, due to the lack of Z direction movement in the center of mass, there was no significant change in the contact force within 1–1.8 s, followed by a left-foot start. In the mid-step gait, there was a significant alternating change in the contact force at the bottom of the feet. During the bipedal support stage of the mid-step, the two decreased and increased, respectively. When the contact force underwent a sudden change, it was the swinging leg landing and hitting the ground that met expectations and fluctuated within a reasonable range.

## 6. Conclusions and Future Work

This paper mainly analyzes the kinematics and dynamics modelling of the mechanical leg of a new hybrid bipedal humanoid robot, and plans and simulates the gait of the robot walking on the ground. The main results are as follows:

(1) Based on the new hybrid humanoid robotic leg, motion analysis was performed and kinematics and dynamics models were established, which enabled the planning of the gait, and laid the foundation for the subsequent application and research of the humanoid robotic leg.
(2) Gait planning of the robot walking on flat ground was performed, and the linear inverted pendulum model was used to plan the trajectory of the robot’s center of mass motion. The joint trajectory of the swinging leg was fitted using a quintic polynomial interpolation method to obtain a stable gait for each stage of the complete walking cycle.
(3) Using MATLAB and Adams to simulate a bipedal robot, the feasibility of the hybrid mechanical leg configuration design and gait planning was verified, laying the foundation for real machine experiments and providing a reference basis for the optimization of subsequent prototype structures, the selection of drive motors, sensor layout, etc.

The proposed gait planning method is suitable for robot walking at low speeds (less than 2–3 km/h). Future research should establish a set of control methods to prevent errors between the reference and actual trajectory from developing, considering the potential effects of unmodeled errors and floor unevenness.

## Figures and Tables

**Table 1:** Table of walking parameters for bipedal robots [18].

Parameter	Value
Single-Step Cycle	0.8 s
Step Distance	0.4 m
Centroid Height	0.89 m
Thigh Length	0.435 m
Calf Length	0.4 m
Maximum Height of the Ankle Joint Center of the Swinging Leg	0.1 m
Support Leg Ankle Joint Center Height above Ground	0.099 m
Distance from Centroid to Hip Joint Height	0.1 m
Distance between Feet	0.2 m

## Data Availability

No new data were created or analyzed in this study. Data sharing is not applicable to this article.
